# CircZNF609 as a prototype to elucidate the biological function of circRNA-mRNA interactions

**DOI:** 10.1080/23723556.2022.2055939

**Published:** 2022-04-11

**Authors:** Manuel Beltran, Francesca Rossi, Irene Bozzoni

**Affiliations:** a Department of Biology and Biotechnology Charles Darwin, Sapienza University of Rome, Rome, Italy; b Center for Life Nano- & Neuro-Science, Fondazione Istituto Italiano di Tecnologia (IIT), Rome, Italy

**Keywords:** RNA-RNA interaction, circRnas, Microtubules, CKAP5, anti-cancer treatments, rhabdomyosarcoma

## Abstract

Circular RNAs (circRNAs) are expressed and are regulated in many biological processes but little is known about their ability to directly control mRNA homeostasis. We show that circRNA zinc finger protein 609 (circZNF609) interacts with several mRNAs increasing the final protein levels, which in the case of the cytoskeleton-associated protein 5 (CKAP5) leads to a stabilized microtubule cytoskeleton and an enhanced tumor cell proliferation.

The latest research showed the importance of RNA molecules as a key factor in the regulatory circuitries. To carry out these fine-tuning activities, RNA not only interacts with proteins and chromatin but often directly pairs with other RNA molecules^.1,2^ In addition to the well-known activity of microRNAs (miRNAs) on mRNAs, much interest is now being directed to the study of how long non-coding RNAs (lncRNAs) control gene expression by targeting specific mRNAs to regulate their stability, translation, and localization by pairwise interaction. Within this frame of research, growing interest is dedicated to circular RNAs (circRNAs), a well-established class of RNA molecules originating from a back-splicing event in which a downstream splice-donor site is joined to an upstream splice-acceptor site, yielding a covalently closed circular RNA. CircRNAs show several peculiar features, such as evolutionary conservation and tissue-specific expression, but above all, they have been found to be deregulated in many pathological conditions, including cancer. Several studies indicate that circRNAs elicit their function as miRNA sponges, but the cell-type-specific microRNA signature restrains this mechanism as a general mode of action. In our work^3^we explored the regulatory roles of the RNA-RNA interactions involving the circRNA zinc finger protein 609 (circZNF609), a circRNA previously reported to be regulated and to control human primary myoblast and embryonal rhabdomyosarcoma (ERMS) cell proliferation^.4,5^ CircZNF609 was also found to be upregulated in several types of human cancers, such as prostate cancer, breast cancer, gastric cancer, nasopharyngeal carcinoma, and hepatocellular carcinoma, where its depletion has been linked with reduced aggressiveness of tumor cells.

Using a psoralen-crosslinking RNA pulldown to detect RNA-RNA interactions *in vivo*, we found that circZNF609 pairs directly with a few mRNAs, among which is the mRNA of the cytoskeleton-associated protein 5 (*CKAP5*). A more detailed molecular study points to the back-splicing junction of circZNF609 as the sequence responsible for this RNA-RNA pairing, underlining the specificity of this interaction in front of the linear *ZNF609* mRNA counterpart. As a result, the *CKAP5* mRNA is stabilized and its translation is enhanced. We demonstrated that this stabilization is produced by ELAV like RNA Binding Protein 1 (ELAVL1, also known as HuR), an RNA-binding protein (RBP) known to induce mRNA stabilization and translation and to control lncRNA metabolism^.6,7^ Specifically, circZNF609 contains several-binding sites for ELAVL1 in its sequence, and the interaction between the circRNA and *CKAP5* mRNA will facilitate ELAVL1 loading from the circular onto the messenger RNA (Figure 1).

**Figure 1.**
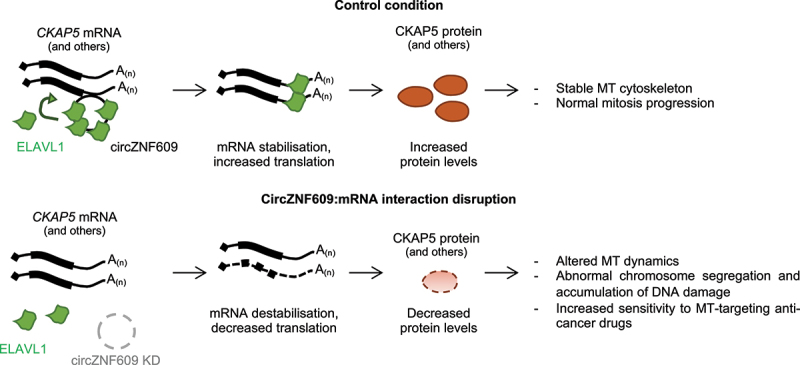
The potential mechanism through which circRNA zinc finger protein 609(circZNF609) regulates mRNA stability. CircZNF609 contains several binding sites for ELAV like RNA binding protein 1 (ELAVL1). The circRNA interaction with the cytoskeleton-associated protein 5 (*CKAP5*) transcript favours the binding of ELAVL1 to the mRNA, increasing mRNA levels and translation. Lack of circZNF609 or disruption of the circZNF609-mRNA interaction will impair mRNAstability and translation of CKAP5 protein leading to altered microtubules (MT)dynamics.

The CKAP5 protein is a highly conserved cytoskeleton-associated factor regulating several aspects of microtubule function: it promotes microtubule nucleation, binds to their growing (“plus”) ends, sustains their elongation and regulates their dynamics^.8^ It also controls mitotic spindle formation and chromosome segregation by stabilizing kinetochore fibers, thus having an important role in mitotic progression^.9^ We demonstrated that the interference of circZNF609, and hence the downregulation of CKAP5 protein levels, produces a de-regulation of microtubule dynamics, leading to abnormal chromosome segregation and DNA damage accumulation, which will eventually produce the cell-cycle halt previously observed in rhabdomyosarcoma^.4^

Microtubule dynamics is essential to all processes depending on the cytoskeleton, including cell migration and differentiation, the building-up of the mitotic scaffold and chromosome segregation. Several first-line chemotherapeutic agents, such as vincristine or Paclitaxel/Taxol, are designed to target either the microtubule assembly from tubulin or their dynamic activity; they are effective chemotherapeutic agents and are still in use to treat cancer of various origins, including rhabdomyosarcoma. We demonstrated that preventing the circZNF609/*CKAP5* mRNA interaction, through either small interfering RNAs (siRNAs) against circZNF609 or locked nucleic acid (LNA)-modified oligonucleotides against the pairing region, sensitizes rhabdomyosarcoma cells to microtubule-targeting chemotherapeutic agents. Moreover, we demonstrated that this regulatory mechanism is also conserved in other cancer cell models such as neuroblastoma and chronic myelogenous leukemia, setting such blockage as a potential therapeutic coadjuvant in cancer treatment.

Microtubule dynamics is essential to all processes depending on the cytoskeleton, including cell migration and differentiation, the building-up of the mitotic scaffold and chromosome segregation. Several first-line chemotherapeutic agents, such as vincristine or Paclitaxel/Taxol, are designed to target either the microtubule assembly from tubulin or their dynamic activity; they are effective chemotherapeutic agents and are still in use to treat cancer of various origins, including rhabdomyosarcoma. We demonstrated that preventing the circZNF609/*CKAP5* mRNA interaction, through either small interfering RNAs (siRNAs) against circZNF609 or locked nucleic acid (LNA)-modified oligonucleotides against the pairing region, sensitizes rhabdomyosarcoma cells to microtubule-targeting chemotherapeutic agents. Moreover, we demonstrated that this regulatory mechanism is also conserved in other cancer cell models such as neuroblastoma and chronic myelogenous leukemia, setting such blockage as a potential therapeutic coadjuvant in cancer treatment.

Microtubule dynamics is essential to all processes depending on the cytoskeleton, including cell migration and differentiation, the building-up of the mitotic scaffold and chromosome segregation. Several first-line chemotherapeutic agents, such as vincristine or Paclitaxel/Taxol, are designed to target either the microtubule assembly from tubulin or their dynamic activity; they are effective chemotherapeutic agents and are still in use to treat cancer of various origins, including rhabdomyosarcoma. We demonstrated that preventing the circZNF609/*CKAP5* mRNA interaction, through either small interfering RNAs (siRNAs) against circZNF609 or locked nucleic acid (LNA)-modified oligonucleotides against the pairing region, sensitizes rhabdomyosarcoma cells to microtubule-targeting chemotherapeutic agents. Moreover, we demonstrated that this regulatory mechanism is also conserved in other cancer cell models such as neuroblastoma and chronic myelogenous leukemia, setting such blockage as a potential therapeutic coadjuvant in cancer treatment.
